# The effects of different amounts of drug microspheres on the vivo and vitro performance of the PLGA/β-TCP scaffold

**DOI:** 10.1080/15685551.2016.1259839

**Published:** 2016-11-28

**Authors:** Liulan Lin, Tianjiang Wang, Qi Zhou, Niandong Qian

**Affiliations:** ^a^ Rapid Manufacture Engineering Center, School of Mechatronic Engineering and Automation, Shanghai University, Shanghai, China; ^b^ Shanghai Institute of Traumatology and Orthopaedics, Ruijin Hospital, Shanghai Jiao Tong University School of Medicine, Shanghai, China

**Keywords:** Different amounts, drug microspheres, osteoinductive, small molecule drugs, vivo experiment

## Abstract

OIC-A006 (BMPs osteogenesis compounds), can stimulate bone marrow mesenchymal stem cells ALP, OPN, OC, Cbfal expression. To stimulate new bone formation in the body. We postulate different amounts of drug microspheres on the PLGA/β-CPT scaffold can produce the effects on performance and sustained release characteristics. In this paper, through adding different amount of carrier drug microsphere, three concentrations scaffolds which are 12.5, 18.75 and 25 μmol/L are prepared by adding different amounts of drug-loaded microspheres. Hereafter called OICM/CPT-200, OICM/CPT-300, OICM/CPT-400. We implant them in rat femur diameter 3 mm depth of 3 mm hole for eight weeks. The degradation, microsphere, delivery properties, with X-ray, micro-CT and histology are tested. Results show that the contain carrier drug microsphere scaffolds become radiopaque, and the gaps between the scaffold and radial cut ends are often invisible. This preliminary study reveals that different carrier drug microsphere has a corresponding effect the performance of stent body, OICM/CPT – 200 scaffolds induction effect is best. Illustrates that the low concentration load OIC-A006 microspheres can promote bone healing, and high concentration of OIC-A006 micro ball is played a inhibitory effect on bone healing process.

## 1. Introduction

China has a large number of patients with bone defects. If they are large bone defects, they can’t completely rely on their own ability to repair, requiring implantation of bionic bone repair materials.[1] An ideal scaffold for bone tissue should be non-toxic, biologically active and biodegradable.[2]

Although autologous bone graft is the best method of treatment of bone defects, the source of autologous bone is limited.[3] Autologous bone is not available for a wide range of bone defects repair.[4] A single type of bone repair materials are generally difficult to meet the requirements of scaffolds for bone tissue engineering.[5] It is a common and effective mean to modify biological materials through certain methods or prepare composite scaffolds by adding other biological materials.[6] Composite scaffolds prepared by this method can effectively improve the performance of bionic bone scaffolds. Therefore, with the application of bone tissue engineering, biomimetic bone used bio-composite materials is the most effective way to solve the problem of bone sources shortage.[7]

Cytokines can modulate innate and adaptive immunity, blood cell production, cell growth, tissue repair and other functions in the bone repair process.[8] Hence, throughout the bone repair process, cytokines is essential. Microspheres loaded cytokine function drugs can reach the goal that drugs participate the whole process of bone repair.[9] It can achieve good results in bone repair because that sustained-release drug-loaded microspheres don’t burst the drug.[10]

The purpose of this paper is to prepare a composite PLGA/β-TCP scaffold with drug carrier microspheres and study drug release properties of drug-loaded composite scaffold to achieve controlled release of drugs. PLGA microspheres which contain drug OIC-A006 are prepared by Ice bath extraction. PLGA/β-TCP scaffolds are prepared by Freeze-drying method. Three concentrations scaffolds are prepared by adding different amounts of drug-loaded microspheres. The scaffolds characteristics of degradation are analyzed. The drug delivery properties of scaffolds are detected by high-performance liquid chromatography (HPLC). A suitable bone repair composite scaffold which drugs can be sustained release can promote the degradation of materials and the in-growth of new bone.

## 2. Materials and methods

### 2.1. Materials

OIC-A006 is donated by the Ruijin Hospital of Shanghai Jiaotong University. Poly (lactic-co-glycolic acid)(PLGA, lactide: glycolide = 85:15, MW 40 000–75 000) is obtained from Sigma Aldrich (Sigma Aldrich, USA). β-TCP is kindly provided by the Shanghai Tissue Engineering Center (China). CH_2_Cl_2_, acetone and vinyl alcohol are obtained from SCRC (SCRC, China).

### 2.2. Preparation of scaffolds

The 0.6 g PLGA is dissolved in 2 ml dichloromethane. The 0.9 g β-TCP is dissolved in 2 ml deionized water. Mix them with stirring. Then, place them in the shock Mills (MM301, Retsch, Germany). Shock those 10 min (oscillation frequency as 15 Hz). Mix the slurry. The 5.787 mg drug OIC-A006 is dissolved in 0.2 ml DMSO. Add them to the mixed slurry. Place them in shock Mills 5 min (concussion frequency of 15 Hz) to obtain a mixed slurry contained drugs.

Drug-loaded microspheres are prepared to take 200, 300, 400 mg, add to the complex drug slurry and placed in shock Mills (MM301, Retsch, Germany) 1 min (concussion frequency of 15 Hz) to prepare different amount of the composite microspheres slurry. At low temperatures, the prepared composite slurry containing the drug microspheres is infused into negative type of bionic bone scaffold in low vacuum mode (temperatures between −15 and 30 °C, pressure 2–2.5 atm).

The negative type with composite slurry containing the drug-loaded microspheres inside is placed in a freeze dryer (LGJ-10D, Beijing Sihuan Technology Co.). After freezing 3 h in low temperature environment (−42 °C), the inside degree of vacuum drop to 18pa in the freeze dryer. After vacuuming once every two hours, the degree of vacuum is maintained between 18 and 30 pa. The temperature is maintained between −42 and −48 °C. Lyophilized 22–24 h, the negative type is removed. The composite bionic bone scaffold contained drugs microspheres obtained a cylindrical shape structure.

### 2.3. Measurement of scaffold porosity

Scaffold porosity is measured by Archimedes’ method according to an established method.[11]

### 2.4. Morphological study

The surface and shape of the OIC-A006-loaded microspheres and scaffolds are observed under a Hitachi SU1510 (Japan) scanning electron microscope (SEM) after gold coating. The microscopic scaffold structure is viewed with the SEM setting at an accelerating voltage of 10 kV.[12] However, owing to the poor electrical conductivity of the OIC-A006 microspheres, set an accelerating voltage of 15 kV to view the microsphere surface.[13]

### 2.5. In vitro degradation of scaffolds

The four groups of scaffolds, eight CPT scaffolds, eight OICM/CPT-200, OICM/CPT-300, OICM/CPT-400 scaffolds, are placed in 50 mL capacity glass bottle. Add 24mLPBS solution which pH value is set to 7.2, the temperature is maintained 37 °C. Detect 4 sets of data each time. Remove in the corresponding period. Then dry the sample using of freeze-drying machine and test the sample.[14]

According to ISO 15,814: 1999, MOD standards, mass losses of the scaffolds in various stages is measured. In accordance with Formula 1, Formula 2, the sample mass loss rate and the retention rate is calculated respectively.[15] The entire test period is 8 weeks, once a week.

(1) $$\text{Quality retention}\;\left(\%\right)=\left(1-\frac{M_{i}-M_{j}}{M_{i}}\right)\times 100\%$$

(2) $$\text{Quality loss rate}\;\left(\%\right)=\left(1-\frac{M_{i}-M_{j}}{M_{i}}\right)\times 100\%$$

where *M*_*i*_ is the mass of the original scaffold (mg), *M*_*j*_ is the mass of dry scaffold after the degradation of *j* weeks (mg).

### 2.6. In vitro drug release studies

OICM/CPT-200, OICM/CPT-300, OICM/CPT-400 scaffolds are prepared. Take them into 24 ml PBS solution. Solution concentration is detected in 1, 3, 7, 14, 28, 42, 56 d using high performance liquid chromatography (HPLC). Each time use 1 mL PBS solution and make up 1 mL PBS solution.[16]

### 2.7. Cell adhesion SEM morphology analysis

Use conventional method to extract the rat bone marrow mesenchymal stem cells (BMSCs).[17] First extracted cells is 0 generation, every digestive cells, cell algebra 1 time. First extracted cells is 0 generation, every digestive cells, cell algebra 1 time. Experiments mainly use relatively stable status between 2 or 3 generation of bone marrow mesenchymal stem cells. Support cell vaccination: add sterilization good material to alpha pre-soaking MEM the culture medium containing 10% FBS.[18] Soak 24 h after abandon to culture, condensed the digestion, counting good cell suspension inoculation on the bracket, the cell suspension inoculation fluid volume is 200 mL, cell number is 10,000/hole, 37 °C, CO_2_ cultivation in the box. Once every 2 days in liquid, training in 4 days and 7 days respectively to detect the cell adhesion.[19]

### 2.8. Measurement of alkaline phosphatase activity

Cellular activity in the scaffold is measured by alkaline phosphatase (ALP) activity assay method.[20] The four groups of scaffolds are placed in 96-well plate and add complete medium containing 10% fetal bovine serum. Pre-incubat for 24 h at 37 °C, 5% CO_2_ incubator. Then, the culture medium is aspirated off completely.[21]

The cells are seeded in the 96 hole of the non-drug-loaded scaffold holes and drug-loaded scaffold holes respectively at the same density.[22] The scaffold pre-incubated 24 h in complete medium before inoculation. Cells are cultured in 37 °C, under the conditions of 5% CO_2_, 100% humidity. After cells culture 1, 3, 7d, the original culture medium is discarded. Each well is added 100μL complete medium and CCK−8–10μL. When cells are incubated in the incubator for 4 h, 100μL liquid is drawn from each well and added into 96 board. Test optical density D value (wavelength = 450 nm) on a microplate reader. ALP activity is measured. Analyze the results.

### 2.9. Animal experiments

The surgical operation and observation of bone growth on scaffolds are assisted by the Animal Care and Experiment Committee of Shanghai Jiao Tong University School of Medicine (No. SYXK 2008–0050). The protocol is approved by the Animal Care and Experiment Committee of the Shanghai Jiao Tong University School of Medicine and University guidelines for the care and use of laboratory animals are followed. Nine mature male Sprague Dawley rats, 24 weeks old, are used as experimental animals. Anesthesia for all animals is induced using 2.5% pentobarbital (30 mg/kg) intrap-eritoneal injection.[23] A critical defect with the size of 3 mm in diameter and 3 mm in depth is created in the end of each rat’s femoral. The defects are filled with scaffolds and the wounds are sutured.

### 2.10. X-ray analysis

At 8 weeks after the scaffolds have been transplanted into the rats, three rats are chosen. X-radiographs are acquired under anesthesia which is induced for all animals by 2.5% pentobarbital (30 mg/kg).[24] The radiograph of the defect site is taken for each rat using a soft X-ray system (Faxitron MX-20, Wheeling, IL, USA). The radiographs are used for qualitative assessment of bone repair.[25]

### 2.11. Micro-computed tomographic analysis

Four groups scaffolds to be included in the 10% of sex of formaldehyde fixed 24 h, in 70% ethanol, then examed using a Micro-CT system. After scanning, a constant volume of interest (VOI) centered over the defect site is selected for analysis of all samples.[26] This VOI is a cylinder with a bottom diameter of approximately 2 mm and a height of approximately 12 mm. Three-dimensional (3D) images are reconstructed based on the VOL. The bone volume fraction (BV/TV,%), trabecular thickness (Tb·Th,μm) and trabecular number (Tb·N/μm^−1^) are calculated using the software provided with the instrument.[27]

### 2.12. Tissue processing

The scaffolds are fixed in 10% neutral formalin for 24 h and placed in 70% ethanol until the examinations are performed. The whole scaffolds is examined with micro-CT. Thereafter, the bone in the defect area is divided into two parts. One part is decalcified, dehydrated and embedded in paraffin. The scaffolds in this part are stained with hematoxylin and eosin (H&E), and examined using light microscopy.[28] Another part is dehydrated, embedded in paraffin for Tartrate-resistant acid phosphatase (TRAP) experiment.

### 2.13. Histological observation

The rats are treated with 5% sodium pentobarbital anesthesia (60 mg/kg). When they are dead, the rats’ humeruses are removed and fixed with 4% formaldehyde solution for 24 h (18–22 °C). The decalcified tissues wash with water for 1–2 h. The tissues are stained with hematoxylin after dewax treatment for 5–10 min. After differentiation with 1% hydrochloric acid alcohol for a few seconds, the tissues are washed with water until fully fixed.[29] Then, eosin staining is observed with neutral rubber. The part stained by H&E is examined using Leica Qwin image-analysis system (Leica Qwin 3.1.0, Bensheim, Germany) to determine new bone formation in the scaffolds.[30]

The rats’ humeruses are washed three times with phosphate buffered saline. At room temperature, fixed liquid is discarded for 30 s, and the humeruses are washed again three times with phosphate buffered saline.[31] The cell culture medium is then air-dried. TRAP solution, which is heated in a water bath (37 °C), is added to the culture wells. Each well contain 250μL TRAP staining solution. The samples are cultured in a water bath for 1 h at 37 °C, after which the TRAP staining solution in the culture wells is discarded.[32] The cells are observed under a fluorescence.

### 2.14. Statistical analysis

Results are expressed as mean ± standard deviation. Statistical significance is determined using analysis of variance (ANOVA). Statistical significance is accepted for *p* < 0.05.

## 3. Results

### 3.1. Scaffold characterization

Contrast scaffolds groups PLGA microspheres which contain drug OIC-A006 are prepared by ice bath extraction. Non-drug comparison blank scaffold and containing different amounts of drug microsphere scaffolds are prepared by Freeze-drying method. As shown in Table 1, it is the four groups corresponding to the drug-loaded scaffolds ratio data.

**Table 1.** Scaffolds’ drug ratio data.

Items	TCP	OICM/CPT-200	OICM/CPT-300	OICM/CPT-400
Drug concentration	0 μmol/L	12.5 μmol/L	18.75 μmol/L	25 μmol/L
Microspheres amount	0 mg	200 mg	300 mg	400 mg
OIC amount	0 mg	0.1841 mg	0.2455 mg	0.3069 mg

### 3.2. Porosity

In this paper, scaffolds’ porosity of four groups are tested. Each group include three scaffolds. All scaffolds apply the same method of manufacturing, the same external conditions and the same ratio of slurry material. The difference is the amounts of drug-loaded microspheres.

The results show that the porosity of scaffolds is from 58.88 to 60.15%. The average porosity of the scaffolds (*N* = 6) is 59.51%. The average porosity of the scaffolds containing drug-loaded microspheres is high than the CPT scaffolds.

### 3.3. Scaffolds degradation

**Figure 1.**
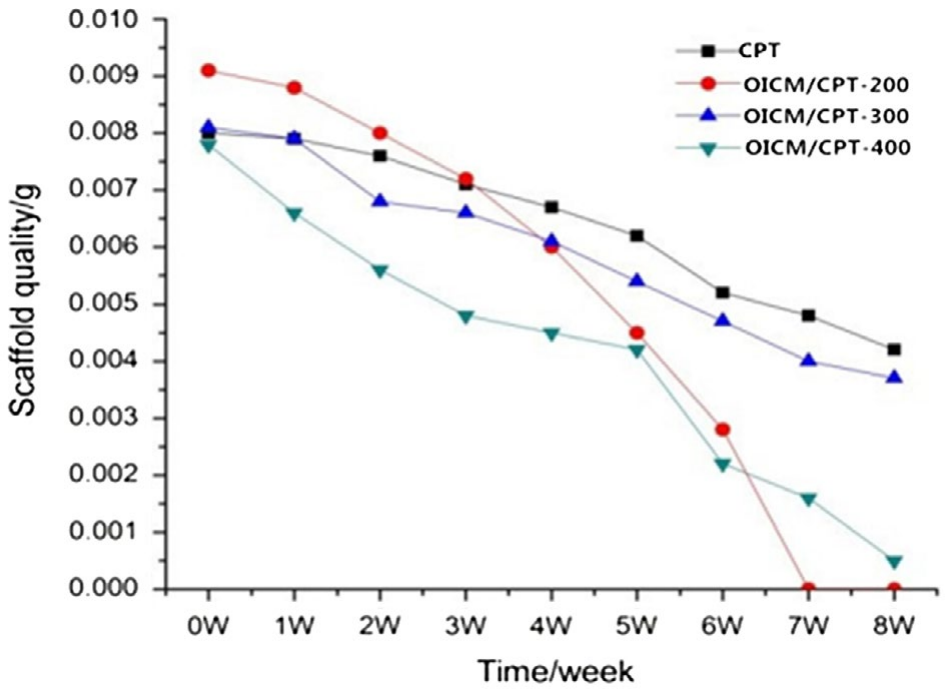
Scaffolds degradation curve.

According to experimental methods and time node, the experimental test data is shown in Figure 1. OICM/CPT-200 scaffold group degradation finish in the seventh week. It is the fastest. The OICM/CPT-300 scaffold group degradation finished in the eighth week. The OICM/CPT-300 scaffold group and blank scaffold group degrade slower. It degrade more than 50% in the eighth week.

Experimental data indicate that adding the drug-loaded microspheres will accelerate the degradation rate of the scaffold. The amount of the microspheres will impact the degradation of the scaffold. The OICM/CPT-200 scaffolds group and OICM/CPT-400 scaffolds group’ degradation rate is 40% faster than OICM/CPT-300 scaffolds group.

### 3.4. Morphology size and structure of the microspheres and scaffolds

**Figure 2.**
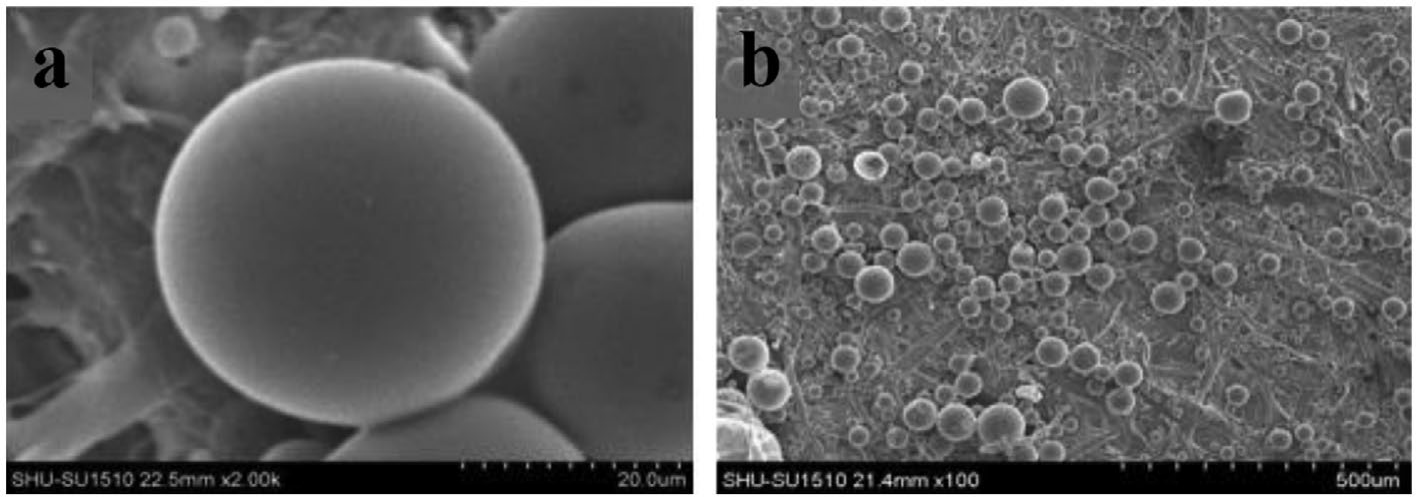
Images of microspheres containing drug OIC-A006 by SEM, (a)×2000, (b)×100.

**Figure 3.**
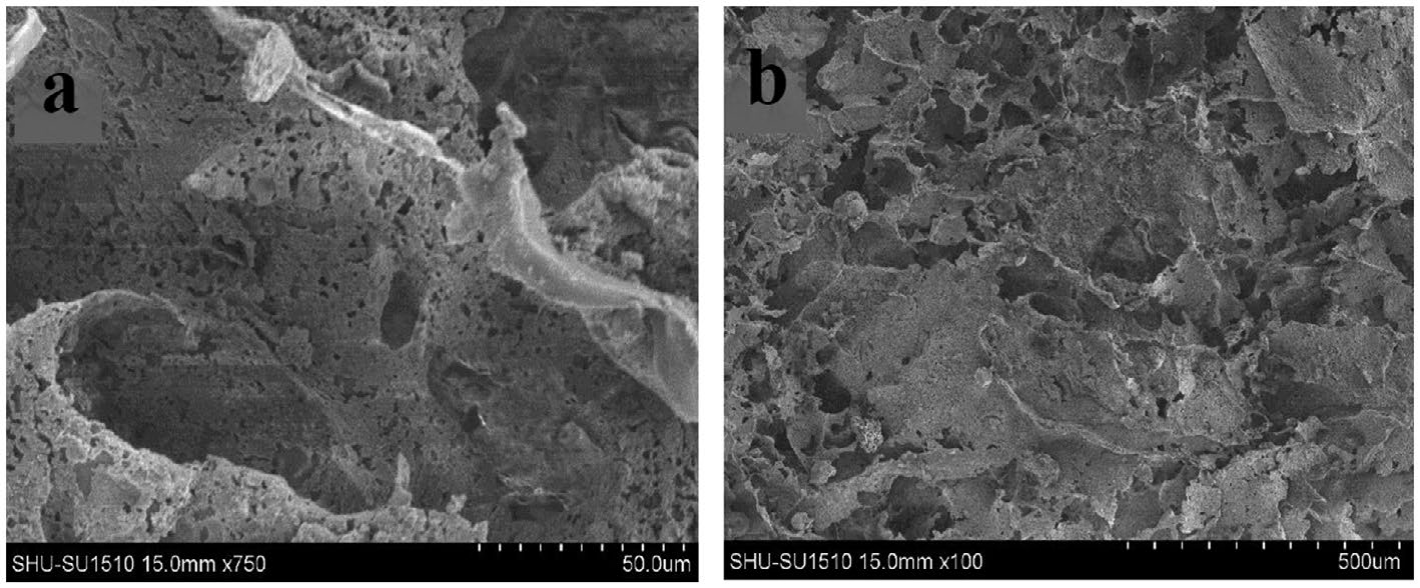
Images of CPT scaffolds by SEM, (a)×750, (b)×100.

The morphology of microspheres and scaffolds are observed using electron microscopy. As shown in Figure 2, the drug-loaded microspheres containing the drug OIC-A006 are prepared by ice bath extraction method. The microspheres which are spherical, smooth, non-porous, an average diameter of microspheres ranging from 20 to 550 μm can be seen from the Figure 2 Since the drug sealed in PLGA microspheres, the release of the drug is determined by the degradation of the microsphere. It can achieve a sustained-release property of the drug. The microstructure of the non-drug-loaded scaffold is shown in Figure 3. The mass ratio of PLGA/β-CPT scaffold is 4: 6. Overall performance of composite scaffold is better. The scaffold is porous, lamellar structure, with good connectivity.[34]

**Figure 4.**
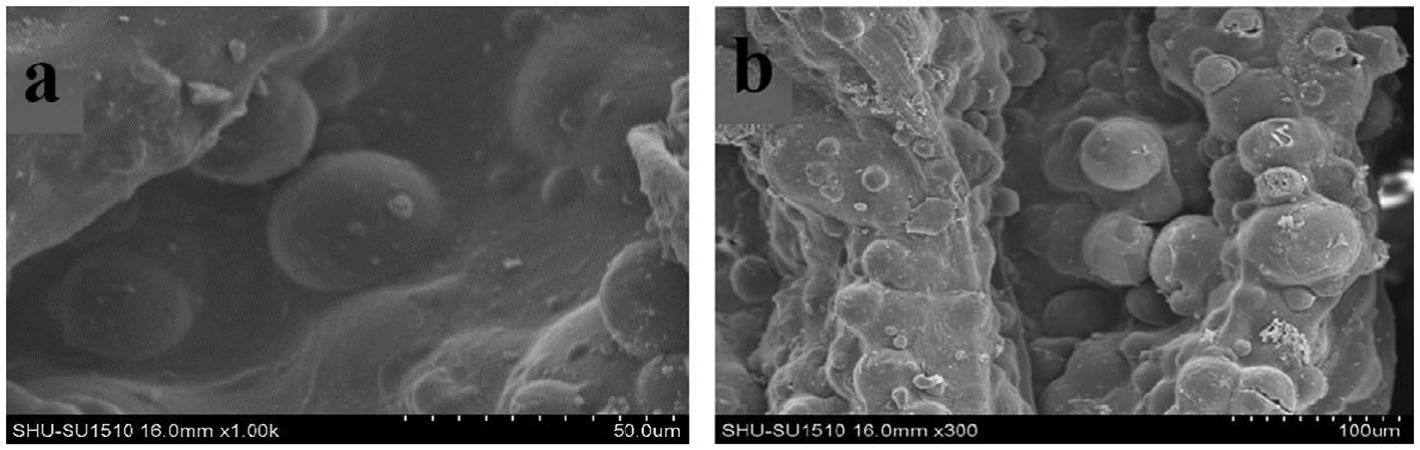
Images of drug-loaded PLGA/β-TCP scaffolds by SEM, (a)×1000, (b)×300.

The microstructure of composite scaffold containing the drug-loaded microspheres is shown in Figure 4. As shown in the figure, the drug-loaded microspheres adhere to the surface of the scaffold. The scaffold’s structure is flaky and porous like the blank. It is the suitable biomimetic scaffolds. The size of scaffold aperture and the drug-loaded microspheres is suitable for adhesion.

### 3.5. Sustained-release characteristic

The scaffold samples are extracted with high-performance liquid chromatography (HPLC) and taken about 15 mg. Then add 3 ml DMSO with temperature oscillation and inject with filter membrane.

**Figure 5.**
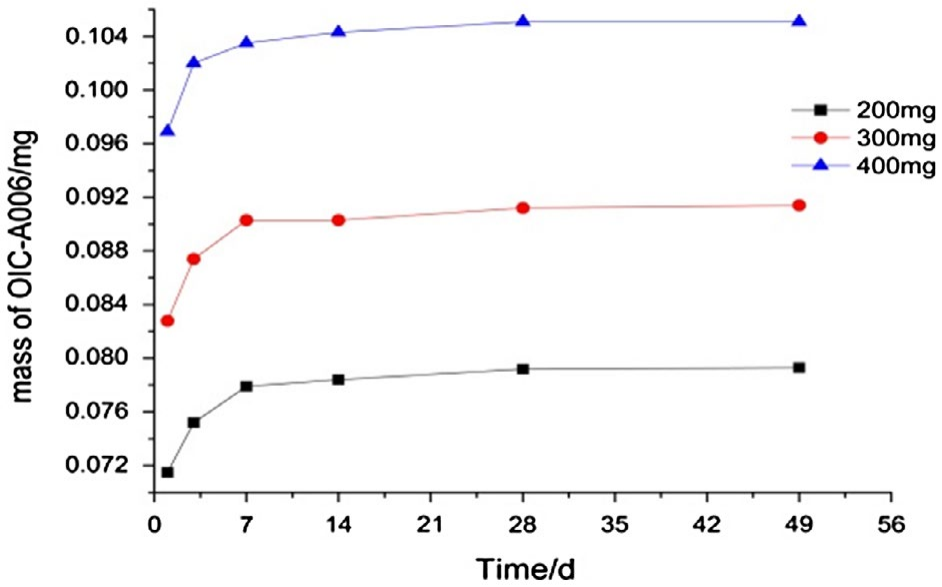
The curve of sustained-release characteristic.

Drug release of different drug-loaded scaffolds groups are obtained by measuring the set point, As shown in Figure 5, it is scaffold drug delivery characteristics for OICM/CPT-200, OICM/CPT-300, OICM/CPT-400 group. As seen from the Figure 5, at the beginning of the experiment, each group of drugs don’t turn out burst phenomenon. It prevents the side effects of overdose.[35] With fast sustained drug release in 13,7 day, OIC-A006 can quickly through the cell membrane, induce bone marrow mesenchymal stem cells, and stimulate the expression of alkaline phosphatase, osteopontin and core osteogenic gene of bone tissue. It can improve bone mass, reach drug release requirements in early bone repair. In 2–8 weeks, due to the presence of pharmaceutical microspheres, the drug begins to slow release.

Pharmaceutical OIC-A006 can significantly promote new bone formation in bone defect site. The whole bone repair process is more smoothly to meet the continuous administration requirements for bone repair. It achieves a controlled release of the drug.

### 3.6. Cell adhesion SEM morphology analysis

**Figure 6.**
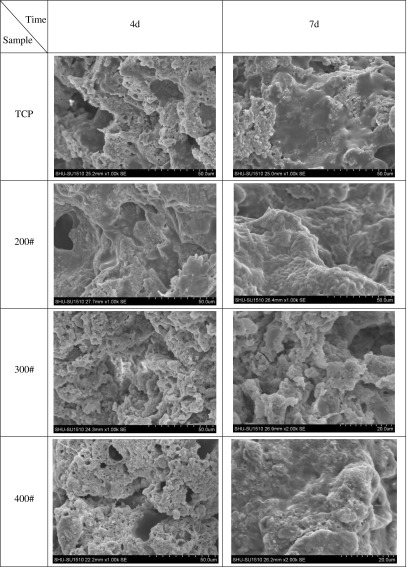
Cell adhesion SEM morphology analysis.

BMSCs on material culture 4 and 7 days respectively, abandon to medium, PBS wash 2.5% glutaraldehyde fixation cells after 15 min, PBS wash after twice, 30, 50, 75, 90 and 75% ethanol gradient dehydration, isoamyl acetate replacement, 37 °C oven drying 4–6 h. After drying of the stent surface BMSCs cell morphology by SEM observation. As shown in Figure 6, as the carrier drug microsphere scaffolds *in vitro* cultivation of cells in 4 d, 7 d after SEM, can be seen from the diagram, OICM/CPT-200 group scaffold cell adhesion proliferation effect is best, stent surface adhesion of cultured cells.

### 3.7. Alkaline phosphatase (ALP) activity

**Figure 7.**
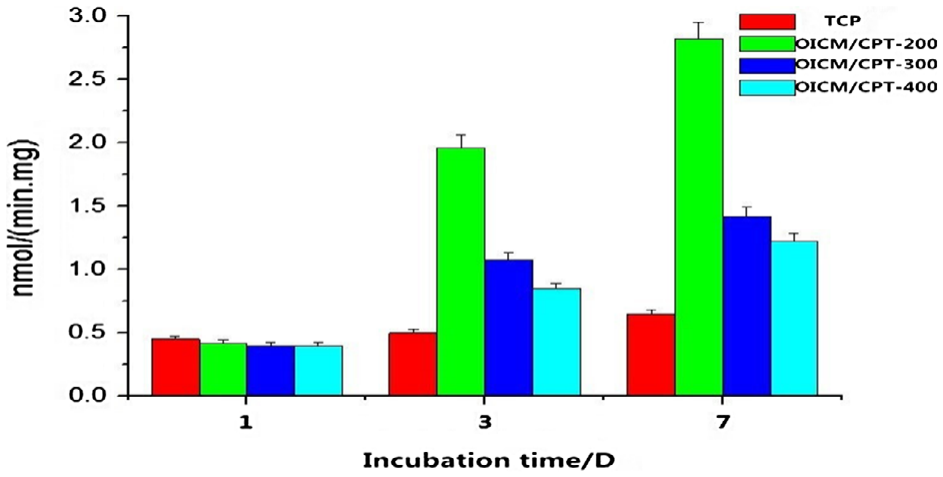
Alkaline phosphatase (ALP) activity test picture.

Alkaline phosphatase activity assay are shown in Figure 7. ALP activity of the drug group is higher than the blank group in the first day, but the difference is small, the effect is not obvious. With the drug-loaded microspheres’ degradation of the drug-loaded scaffold, the drug releases and increases the ALP activity. It shows a strong osteoinductive capacity. Data shows that the ALP activity of OICM/CPT-200 is higher than the blank group and other groups. Qualitative analysis of ALP activity shows that the composite scaffold containing with drug-loaded microspheres has a better osteogenic differentiation than the blank one.

### 3.8. Radiographic examination

**Figure 8.**
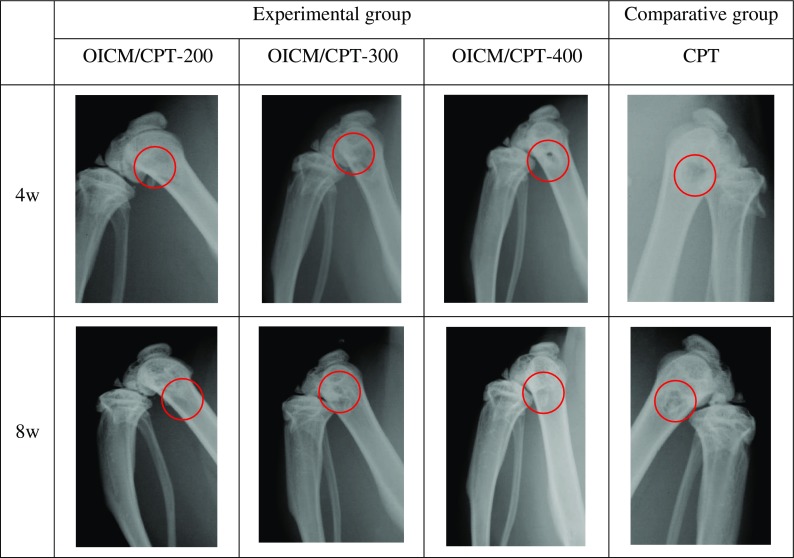
X-radiographs of rats at 4, 8 weeks. Scaffolds with CPT, OIC/TCP–200, OIC/TCP–300 and OIC/TCP-400.

Figure 8 shows X radiographs of the rats at the 4th and 8th week. Along with the growth of the time, 4 weeks after the TCP and experimental group scaffold material has not been clearly saw a callus formation. 8 weeks after the scaffold covered stent surface callus, new bone scabs distribution is uniform, extending from peripheral to the central defect parts, and the control group with a small amount of new bone callus appeared in the defect areas surrounding .The experimental results, as shown in the Figure 8, X-ray shows as the growth of the time, four groups of implant stents have been degraded. Four groups of stents, OIC/TCP-200 groups of stents effect is better than the rest of the group and the blank control group, the bone defect have been full of new bone, the X-ray shows a very good repair effect. The rest of the group of stents in the bone defect has been gradually degradation and new bone in-growth.

### 3.9. Micro-CT analysis

**Figure 9.**
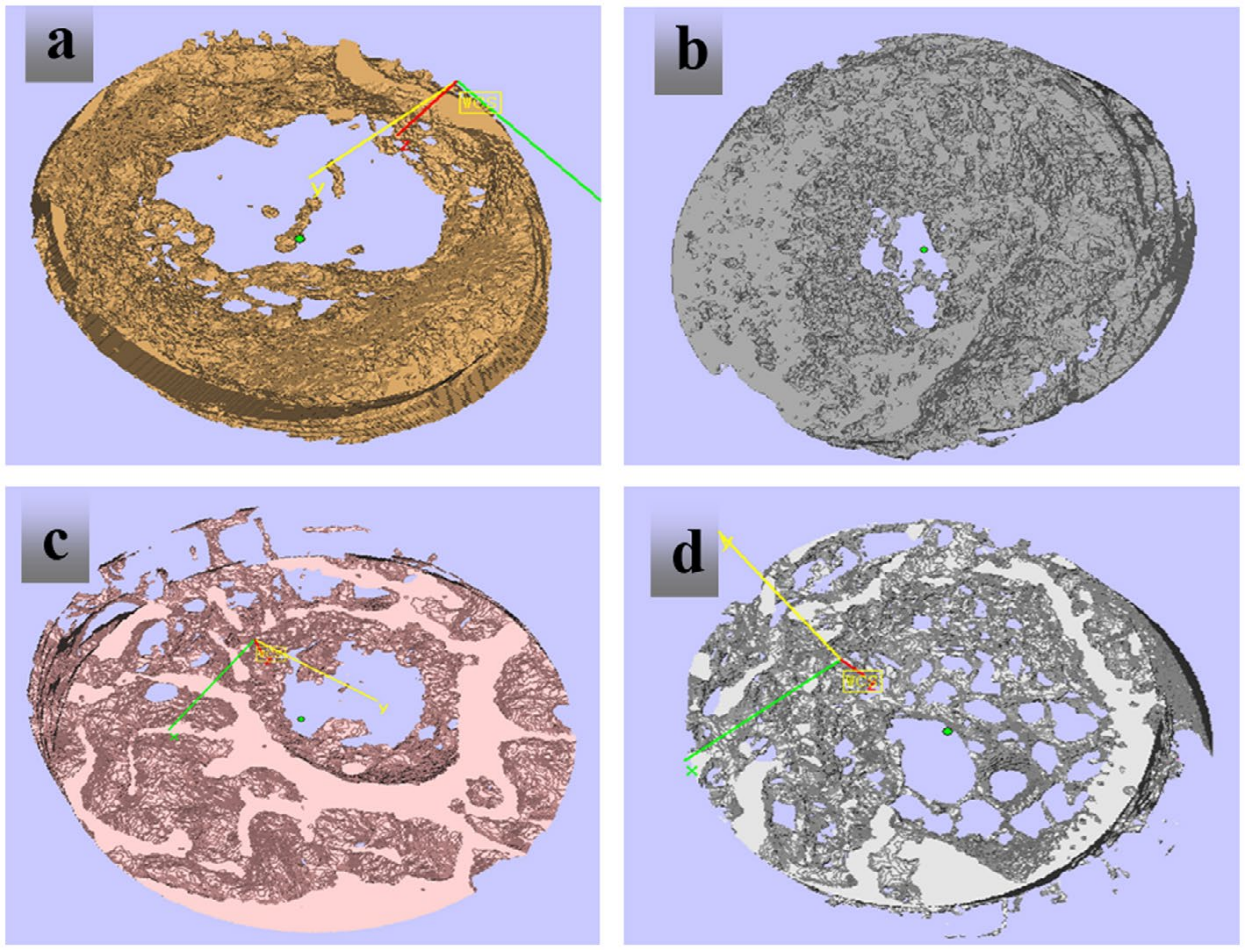
Micro-CT images at 8 weeks, (a) CPT, (b) OIC/TCP–200, (c) OIC/TCP–300, (d) OIC/TCP–400.

Since the organic matrix in the PLGA/β-CPT scaffold is removed during processing, the degree of mineralization of PLGA/β-CPT scaffold is significantly higher than that in normal and newly formed bone. Therefore, it is easy to distinguish the CPT scaffold from new bone by different grayscales in 2D and 3D images.[35] Eight weeks after surgery, there are only small amounts of new bone in the pores of the implanted CPT scaffolds (Figure 9(a)). There are moderate amounts of new bone in the pores of the implanted OICM/CPT scaffold (Figure 9(b)). However, the CPT trabeculae in the OICM/CPT-200 scaffold are significantly decreased and pores in the scaffold are almost filled with new bone that showed darker grayscale (Figure 9(c)). The trabecular meshwork is invisible in the new bone area.

**Figure 10.**
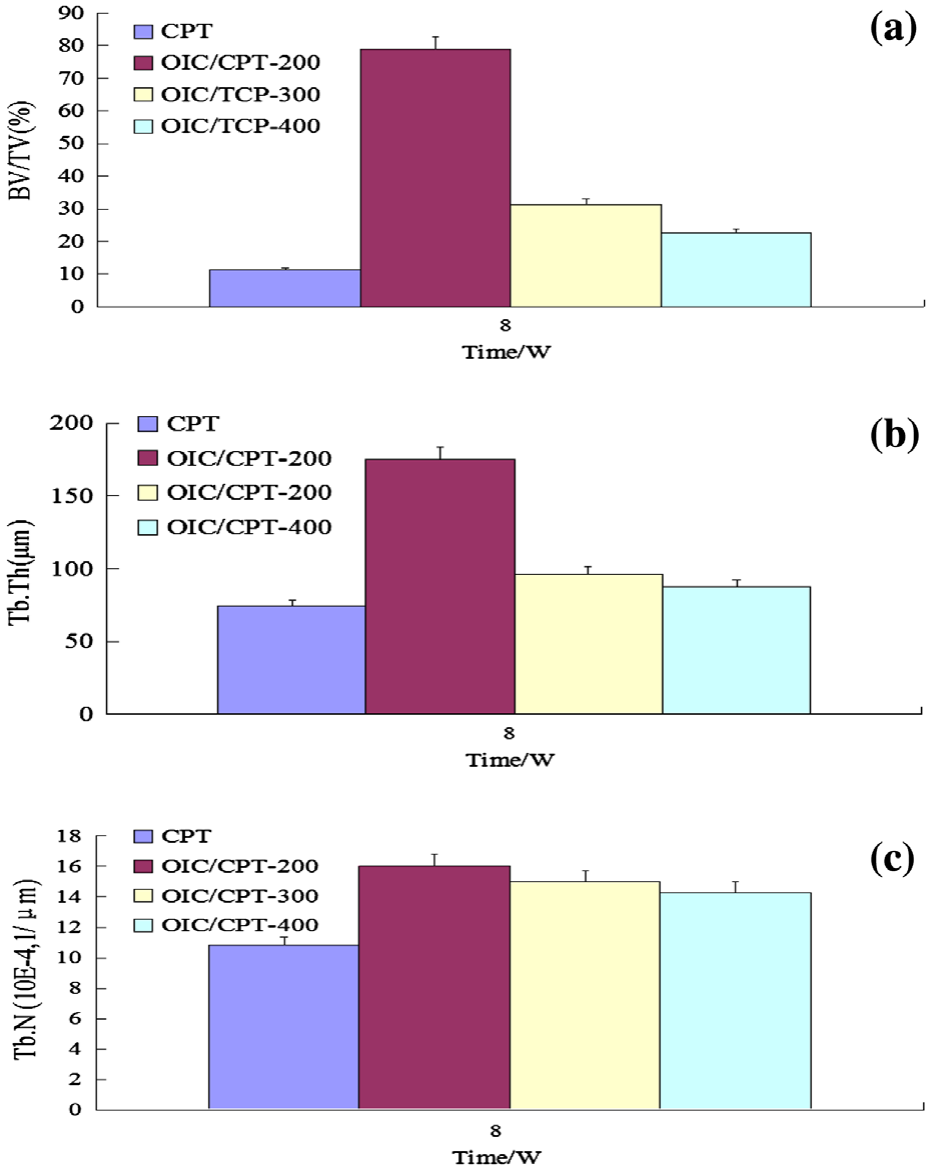
Comparison of BV/TV (a), Tb·Th (b) and Tb·N (c) between 8-week implanted CPT, 8-week implanted OICM/CPT-200, 8-week implanted OICM/CPT-400.

Compare with CPT scaffolds, the porosity and pore size show at the 3-D level are significantly decreased in OICM/CPT-200, OICM/CPT-300 and OICM/CPT-400 scaffolds after 8 weeks of implantation .The cortical-like bone structure could be identified on the surface of the OICM/CPT-200 scaffold. The quantitative data demonstrate that BV/TV (Figure 10(a)), Tb·Th (Figure 10(b)) and Tb·N (Figure 10(c)) in CPT scaffolds are significantly lower than that in OICM/CPT-200, OICM/CPT-300 and OICM/CPT-400 scaffolds implanted for 8 weeks (*p* < 0.05). For the latter three groups, BV/TV and Tb·Th in the OICM/CPT-200 scaffold are significantly greater than that in the CPT scaffold but Tb·N do not show a significant difference. Gaps are often visualized between the implant and radial cut ends in the OICM/CPT-300 and OICM/CPT-400 groups, but not obvious in the OICM/CPT-200.

### 3.10. Histology

**Figure 11.**
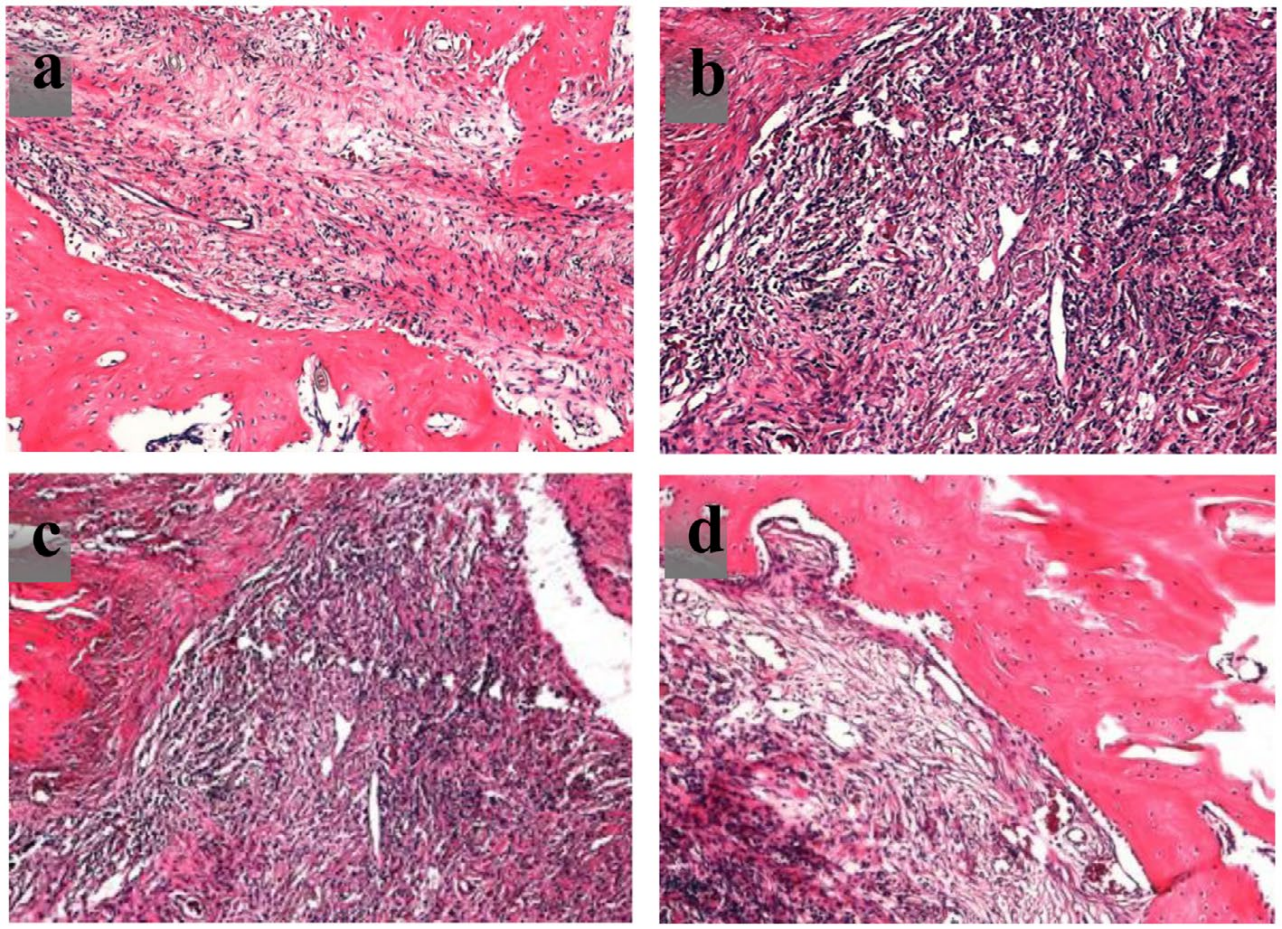
H&E staining at 8 weeks for scaffold with (a) CPT, (b) OICM/CPT-200, (c) OICM/CPT-300, (d) OICM/CPT-400.

Bone formation performance in the rat bone defects is analyzed by hematoxylin and eosin (H&E) staining after eight weeks. As shown in Figure 11, a large number of osteoblasts scaffold and there is new bone growth in the bone defect.

It showed that some pores in implanted CPT scaffolds are filled with new bone, but many material remnants are also present (Figure 11(a)). Compare with the CPT group, there are considerably increased new bone and decreased material remnants in OICM/CPT scaffolds. Large masses of new bone coalescence of new bone formed in different pores after the bionic material are absorbed. In addition, many cavities in the new bone area in the OICM/CPT scaffold are occupied by bone marrow elements. The bone repair for the OICM/CPT-200 scaffold is best. The degradation time of scaffold materials *in vivo* is equal to that observed for the degradation experiment *in vitro*.

**Figure 12.**
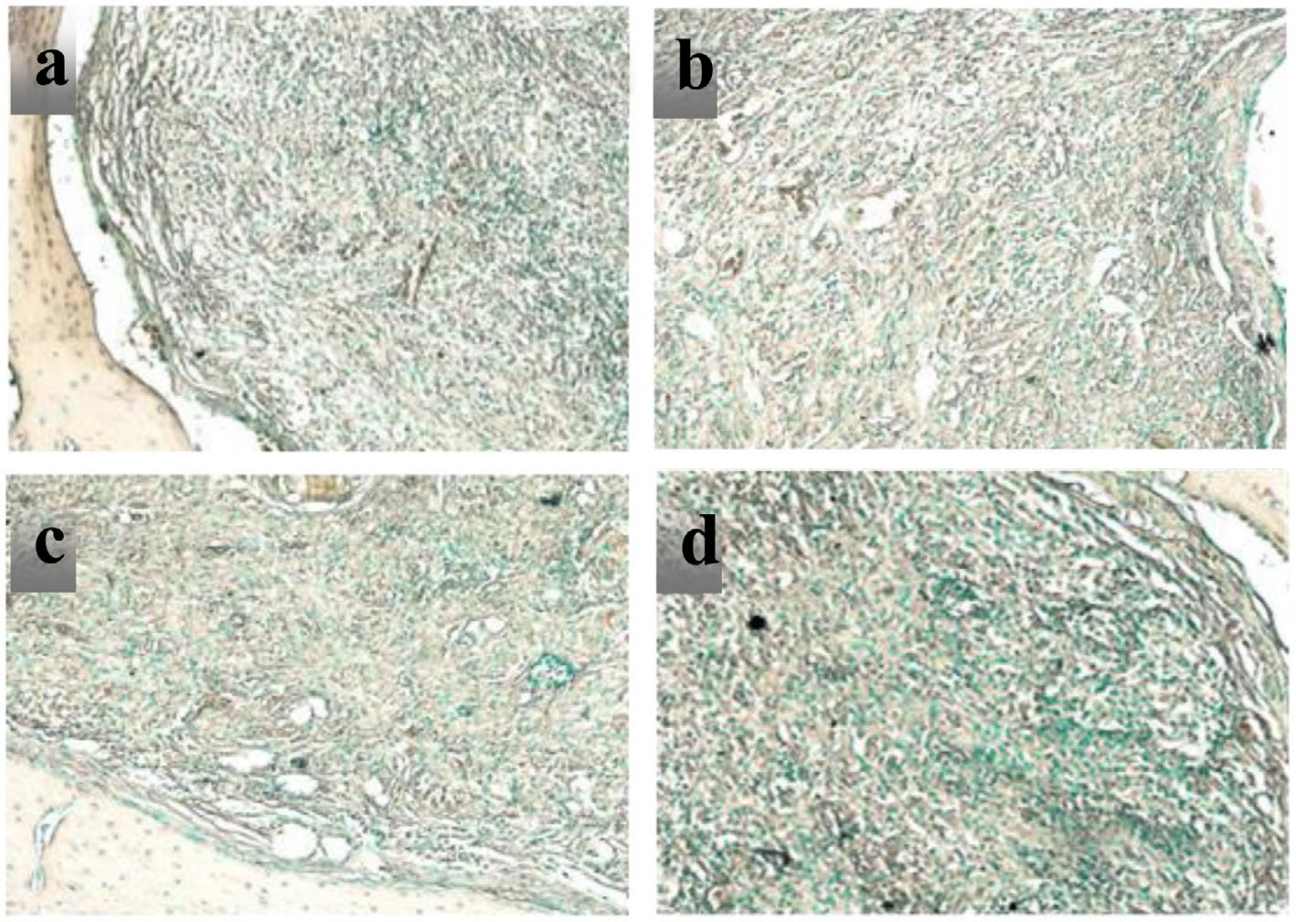
TRAP staining at 8 weeks for scaffolds with (a) CPT, (b) OICM/CPT-200, (c) OICM/CPT-300, (d) OICM/CPT-400.

In the 8th week, humeruses of the rats are removed for a TRAP experiment. As shown in Figure 12, a large number of osteoclasts are gathered in the interior of CPT, OICM/CPT-200, OICM/CPT-300 and OICM/CPT-400 scaffold. The material remnants are easily distinguished from new bone by the different staining intensity and morphology (Figure 12). The remaining material had no osteocyte lacunar profiles (Figure 12(a)).

However, the percent of new bone is significantly greater in OICM/CPT scaffolds (Figure 12(b)–(d)) than in pure CPT scaffold at 8 weeks (Figure 12). The new bone is contained numerous osteocyte profiles (Figure 12(c)). The bone repair effect of OICM/CPT-200 scaffold is best. Owing to the large numbers of osteoblasts and osteoclasts, the new bone grew into scaffolds and the scaffold had been degraded.

## 4. Discussions

### 4.1. Microspheres and scaffolds

In this paper, PLGA microspheres which contain drug OIC-A006 are prepared by Ice bath extraction. As shown in Figure 5, they are SEM images of drug-loaded microspheres. Diameter of microspheres are predominantly in 20–500 μm. Experimental studies have shown that the higher magnetic stirring speed, the longer the time, the microspheres more uniform. Suitable ice bath environment can improve microspheres forming rate.[33]

CPT scaffold is prepared by freeze-drying technology. The SEM images of scaffold are shown in Figure 6. There is a clear lamellar structure. Porosity have connectivity. Pore size is 20–500 μm. Average porosity is about 59%. In the preparation process, the pre-freezing temperature plays an important role in pore structure and porosity of the scaffold, it also has some influence on the mechanical strength of the scaffold.[34] The lower pre-frozen temperature decreases the porosity of the scaffold, increases the strength of the scaffold.[35] Conversely, the higher the pre-frozen temperature, the greater the porosity of the scaffold, the lower the strength of the scaffold. In this paper, in order to obtain a suitable porosity and mechanical strength, scaffolds is manufactured with a pre-frozen temperature of −42 °C. Using the above method, drug-loaded scaffolds are prepared by adding different amounts of the drug microspheres. Adding drug microspheres have little impact on the porosity of the scaffolds. The mechanical strength of the scaffolds decreases slightly. It meets the biological scaffolds’ strength requirements. The images of prepared OICM/TCP scaffold by SEM are shown in Figure 7. It has appropriate porosity and a suitable environment for cell in-growth, adhesion, proliferation. Microspheres adhere to scaffold’s porosity. It provides a suitable conditions for sustained release of the drug.

### 4.2. Performance of drug-loaded scaffolds

Three groups of drug-loaded scaffolds, OICM/CPT-200, OICM/CPT-300, OICM/CPT-400 are prepared by adding different amounts of Drug-loaded microspheres. In the degradation test, the scaffold of OICM/TCP-200 group degraded fastest. It degraded in first seven weeks. The scaffold of OICM/TCP-400 group degraded Secondly. It degraded in the eighth week. The scaffolds of OICM/TCP-300 group and the blank group degraded slowest. The degradation of it reached half in the eighth week.

### 4.3. Drug release characteristics

Drug OIC-A006, as a kind of BMP2 function, plays a role in inducing bone repair in the bone repair process. Adding drug OIC-A006 to biomimetic scaffold can improve scaffold osteoinductivity. It can make the bone repair process more smoothly. In general dosing process, drugs will be suddenly released. In the initial scaffold implantation, excessive drug release will produce toxic side effects on cells on bone defect.[36]

To prevent the burst effect of the drug, drug-loaded microspheres are added to the scaffolds. As shown in Figure 7, The drug coated in microspheres to prevent violent drug release. Microsphere size determines the release time of the drug. The smaller the size of the microspheres, the faster the drug release.

Drug release profiles showed that the joined drug microspheres realize sustained release of the drug. In the early degradation of 1–7d, the drug of OICM/TCP-200, OICM/TCP-300, OICM/TCP-400 groups did not occur burst. In the 2–7 W, the drug released slowly to maintain a sustained release of cytokine function drug in the bone repair region. The whole bone repair process is smooth. It achieved a controlled drug release in the whole process.

### 4.4. ALP activity

ALP activity of 4 scaffolds groups is detected. Quantitative determination of ALP activity analysis showed that the drug-loaded groups are higher than the blank group in the first day, but the gap is small. The reason is the initial drug release. With the degradation of drug-loaded scaffolds containing the drug-loaded microspheres, the drug subsequently release. The data shows that the ALP activity increase rapidly. It shows a strong osteoinductive capacity. Data shows that the ALP activity of the OICM/TCP-200 is higher than the blank group and the other groups. It indicates that there are more ALP enzyme produced from bone marrow stromal cells. The ALP activity of the OICM/TCP-300 is lower than the OICM/TCP-200. It is probably for the slower degradation rate of the scaffold, the lower drug release quantity. The ALP activity of the OICM/TCP-400 is lower than the OICM/TCP-200. It is probably for the high dose of the drug. Due to the presence of OIC-A006 drug micro-side-effects, s the ALP activity of OICM/TCP-400 is lower than OICM/TCP-200. In short, ALP qualitative analysis shows that the composite scaffold containing drug-loaded microspheres has a good osteogenic differentiation.

### 4.5. In-vivo implant test

Stents are implanted into the rat experiment is carried out, for X-ray test, MicroCT, HE dyeing experiments, TRAP dyeing experiments, etc. Bracket of X-Ray test shows that the experimental group had obvious callus formation, illustrate the OIC-A006 can effectively stimulate new bone formation; MicroCT experiments showed that three sets of stent have a large number of new bone formation, OIC/CPT-200 more effective s for new bone formation; Also, HE dyeing experiments, the TRAP experiments showed bone defect with new bone formation, defect exist a large number of osteoclasts, and osteoblasts, as the degradation of stents, new bone gradually generated, the release of drug OIC-A006, promoted the formation of new bone, These phenomena shows that drug stents and don’t take medicine to induce new bone formation process is different. Early carrier drug microsphere drug release of three groups of stents can make the OIC-A006 quick through the cell membrane, inducing bone marrow mesenchymal stem cells to differentiate between, alkaline phosphatase, osteopontin and core factors, such as combination of bony landmark gene expression, and can improve bone tissue bone mass, to fulfill the requirements of the drug release in the early bone repair. Drug sustained release can obviously promote the new bone formation, in the process of new bone formation, comparative analysis, the OIC/CPT-300 stents due to slow degradation, drug release quantity relative to the OIC/CPT-200, OIC/CPT-400 is slow, the OIC/CPT-400 stents due to drug dosage on the high side, the side effects of inhibiting the bony landmark gene expression, considering various tests *in vivo* and *in vitro*, OIC/CPT-200 scaffold in the best performance, osteogenesis effect is best.

## 5. Conclusions

By adding different amount of three groups stent carrier drug microsphere, respectively to produce the OIC/TCP-200, OIC/TCP-300, OIC/TCP-400 scaffold, three groups scaffolds with CPT experiment contrast, through the degradation test, cell adhesion test, drug test and ALP activity test results show that the OIC/TCP-200 scaffold *in vitro* comprehensive performance is best. X-ray testing, MicroCT experiment, HE staining, the TRAP dyeing experiment shows the three groups of drug-loading stents compared with the blank has new bone formation effect, the OIC/TCP-200 *in vivo* performance better, and *in vitro* performance form. Illustrates the low concentration load OIC microspheres can promote bone healing, and high concentration of OIC micro ball is played a inhibitory effect on bone healing process.

## Disclosure statement

No potential conflict of interest was reported by the authors.

## Funding

This work was supported by the Natural Science Foundation of China (NSFC) grant funded by the Chinese government [grant number 81201386].
